# Effects of different intermittent pneumatic compression stimuli on ankle dorsiflexion range of motion

**DOI:** 10.3389/fphys.2022.1054806

**Published:** 2022-11-23

**Authors:** Takuma Yanaoka, Urara Numata, Kanna Nagano, Shiho Kurosaka, Hiroki Kawashima

**Affiliations:** ^1^ Graduate School of Humanities and Social Sciences, Hiroshima University, Hiroshima, Japan; ^2^ Linear R&D Department SectionⅡ, Nitto Kohki Co., Ltd., Tokyo, Japan

**Keywords:** massage, weight-bearing lunge test, artery blood flow, pressure-to-pain threshold, muscle hardness, heart rate variability, intermittent pneumatic compression (IPC)

## Abstract

Despite substantial evidence of the effectiveness of intermittent pneumatic compression (IPC) treatments for range of motion (ROM) improvement, little evidence is available regarding how different IPC stimuli affect ankle dorsiflexion (DF) ROM. This study aimed to investigate the effects of different IPC stimuli on the ankle DF ROM. Fourteen, university intermittent team sport male athletes (age: 21 ± 1 year, height: 1.74 ± 0.05 m, body mass: 70.9 ± 7.7 kg, body fat percentage: 14.2 ± 3.6%, body mass index: 23.5 ± 2.5 kg/m^2^; mean ± standard deviation) completed four experimental trials in a random order: 1) no compression with wearing IPC devices (SHAM), 2) the sequential compression at approximately 80 mmHg (SQUEE80), 3) the uniform compression at approximately 80 mmHg (BOOST80), and 4) the uniform compression at approximately 135 mmHg (BOOST135). For the experimental trials, the participants were initially at rest for 10 min and then assigned to either a 30-min SHAM, SQUEE80, BOOST80, or BOOST135. Participants rested for 20 min after IPC treatment. The Weight-Bearing Lunge Test (WBLT), popliteal artery blood flow, pressure-to-pain threshold (PPT), muscle hardness, heart rate variability, and perceived relaxation were measured before (Pre) and immediately after IPC treatment (Post-0) and 20 min after IPC treatment (Post-20), and the changes in all variables from Pre (Δ) were calculated. ΔWBLT performance, ΔPPT, and Δperceived relaxation in all IPC treatments were significantly higher than those in SHAM at Post-0 and Post-20 (*p* < 0.05). ΔPopliteal artery blood flow in BOOST80 and BOOST135 was significantly higher than that in SHAM and SQUEE80 at Post-0 (*p* < 0.05). ΔMuscle hardness and Δheart rate variability did not differ significantly between trials. In conclusion, IPC treatments, irrespective of applied pressure and mode of compression, increased ankle DF ROM. This resulted from decreased pain sensitivity (i.e., increased PPT). In addition, high inflation pressure and frequency did not provide additional benefits in increasing ankle DF ROM.

## 1 Introduction

Ankle dorsiflexion (DF) range of motion (ROM) is associated with the risk of a wide variety of lower extremity injuries in athletes (Mason-Mackay et al., 2017). Restricted ankle DF ROM limits the ability to pass the leg forward over the foot and to lower the center of mass during squat-type movements (Piva et al., 2005). This may be compensated by subtalar and midfoot pronation or valgus of the knee (Piva et al., 2005), which are associated with chronic and acute injuries (Mason-Mackay et al., 2017). Furthermore, restricted ankle DF ROM and associated reductions in hip and knee flexion at landing increase the loading rate and ground reaction forces, thereby increasing the risk of injury (Mason-Mackay et al., 2017; Brazier et al., 2019). Ankle DF ROM is associated not only with the risk of injuries, but also with sports performance. Previous studies have shown that restricted ankle DF ROM decreases changes in directional capability (Gonzalo-Skok et al., 2015) and unilateral dynamic balance (López-Valenciano et al., 2019), both of which are key skills in intermittent team sports. Therefore, strategies to maintain and improve ankle DF ROM are important for athletes.

Intermittent pneumatic compression (IPC) may be one such treatment approach. The IPC treatment utilizes whole-leg sleeves that operate by inflating and deflating a series of zones at selected pressures. Historically, IPC has been employed in clinical settings to combat vascular diseases by increasing the blood flow (Kumar and Walker, 2002). IPC has been shown to improve lower-limb artery hemodynamics (Sheldon et al., 2012), resulting from: 1) an increase in the arteriovenous pressure gradient secondary to venous emptying (Delis et al., 2000) and 2) an improvement in endothelial function by the production of endothelial vasodilators (i.e., nitric oxide and vascular endothelial growth factor) in response to an increase in shear stress (Sheldon et al., 2012). In addition, evidence regarding IPC in an athletic setting has recently increased. Previous studies have reported that IPC has little to no benefits in a 4-min cycling time-trial (Overmayer and Driller, 2018), a 30-s Wingate anaerobic test (Martin et al., 2015b), muscle glycogen synthesis (Keck et al., 2015), and autonomic system recovery (Valenzuela et al., 2018). However, some investigations have indicated that IPC increased flexibility (Sands et al., 2014; Haun et al., 2017b), pressure-to-pain threshold (PPT) (Sands et al., 2015; Haun et al., 2017b), and blood lactate clearance (Martin et al., 2015b) and reduced skeletal muscle oxidative stress and proteolysis markers (Haun et al., 2017a; Haun et al., 2017b). Potential mechanisms contributing to increased ROM following massage, including IPC, are quite diverse. For example, decreased pain sensitivity (i.e., an increase in PPT) of connective tissue may improve stretch tolerance and thus increase ROM (Macdonald et al., 2014). Improved hemodynamics may induce warming and thixotropic effects *via* neural feedback mechanisms (Behm and Wilke, 2019). Moreover, a variety of receptors (e.g., Ruffini cylinders and Pacinian corpuscles) respond to massage pressure, which may affect parasympathetic activation, resulting in relaxation of muscles (i.e., reduced muscle hardness) (Behm and Wilke, 2019).

Despite substantial evidence of the effectiveness of IPC treatment for ROM improvement, there is little information regarding the appropriate setting of IPC treatment (i.e., applied pressure and mode of compression) for ROM improvements. Various pressures and modes of compression are commercially available; therefore, they are currently used in clinical and athletic settings. It is important to address this issue because clinicians and athletic trainers are challenged by the task of providing evidence-based recommendations regarding IPC treatment. A previous study investigated the impact of different IPC stimuli (frequency: two to four impulses per min; applied pressure: 60–140 mmHg; mode: IPC of the foot, calf, and both foot and calf) for lower-limb venous emptying (Delis et al., 2000). This study showed that foot and calf compression with higher frequency and applied pressure caused improvements in lower-limb venous emptying and the resultant increase in arterial blood flow (Delis et al., 2000, 2001). As noted above, an improvement in hemodynamics is one of the potential mechanisms contributing to increased ROM following massage. Thus, the applied pressure and mode of compression in IPC treatment may cause an increase in ROM.

Therefore, the present study aimed to investigate the effects of different IPC stimuli on ankle DF ROM. We hypothesized that 1) IPC, irrespective of applied pressure and mode of compression, would increase ankle DF ROM resulting from increased artery blood flow and PPT, and 2) the magnitude of improvement in ROM would be high under high pressure and frequency concurrent massage due to a greater increment in artery blood flow under high pressure and frequency massage compared to low pressure and frequency massage.

## 2 Materials and methods

### 2.1 Participants

Fourteen, university intermittent team sport male athletes participated in this study (age: 21 ± 1 year, height: 1.74 ± 0.05 m, body mass: 70.9 ± 7.7 kg, body fat percentage: 14.2 ± 3.6%, body mass index: 23.5 ± 2.5 kg/m^2^; mean ± standard deviation [SD]). A power calculation using ROM data from a previous study (Sands et al., 2014) was performed using a calculated effect size (partial η^2^) of 0.42, *α* = 0.05, and *β* = 0.20, which determined that eight participants were required to demonstrate a difference in ROM after IPC treatments. The participants did not have a history of lower extremity injury within 6 months before the study and were free of cardiovascular and peripheral vascular disease at the time of the study. This study was approved by the Ethics in Human Research Committee of Hiroshima University (Approval number: 2020043), and all participants provided written informed consent to participate in this study.

### 2.2 Experimental design

All participants completed a preliminary visit to determine their physical characteristics and to perform a familiarization trial before completing four experimental trials (1: no compression while wearing IPC devices [SHAM], 2: IPC treatment using sequential compression (commercial name: Squeeze) with an applied pressure of approximately 80 mmHg [SQUEE80], 3: IPC treatment using uniform compression (commercial name: Boost) with an applied pressure of approximately 80 mmHg [BOOST80], 4: IPC treatment using uniform compression with an applied pressure of approximately 135 mmHg [BOOST135]) using a randomized cross-over design.

All trials were separated by at least 3 days and were performed at the same time of day for each participant to avoid any circadian rhythm-related variations. Participants refrained from consuming alcohol and caffeine for 24 h prior to each experimental trial and fasted for 3 h, except for the consumption of water, before each experimental trial. The participants were asked to avoid altering their regular lifestyle habits, exercise, and diet throughout the study. All the trials were performed during the competitive season.

### 2.3 Experimental procedure

During the preliminary visit, body weight and body fat percentage were measured using multifrequency bioelectrical impedance analysis equipment (InBody 470, InBody Japan, Japan) (Alves et al., 2014). For familiarization, 30-min IPC treatment using BOOST135 was performed.

In the four experimental trials, participants were initially at rest for 10 min and then wore an IPC device (Doctor Medomer DM-4S, Nitto Kohki, Japan). Participants were then assigned to either a 30-min SHAM, SQUEE80, BOOST80, or BOOST135. Participants rested for 20 min after IPC treatment. The participants remained in the supine position throughout the study except for the measurements. During this experiment, the participants were only allowed to read books and were prohibited from sleeping, using computers, or drinking water.

### 2.4 IPC treatments

**FIGURE 1:**
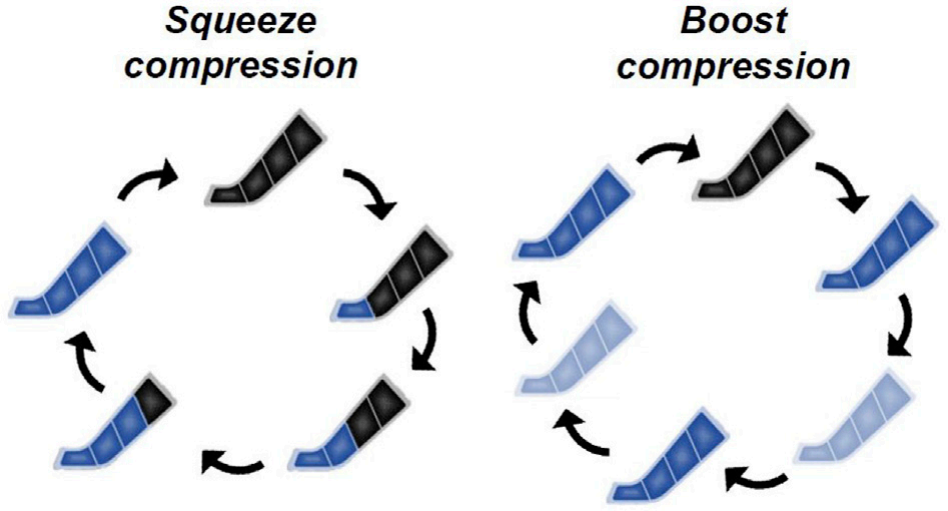
Representative image of inflation protocols in intermittent pneumatic compression (IPC) stimuli.

The IPC stimuli are shown in Figure 1. The massage device consisted of two leg sleeves that contained four circumferential inflatable chambers encompassing the leg from the foot to the distal thigh. The leg sleeve was connected to an automatic pneumatic pump and controlled the target inflation pressure and duty cycle of each chamber. The target pressure and duty cycle were pre-programmed and commercially available. We employed the applied pressure at 80 or 135 mmHg since 1) the applied pressure at 80 mmHg was employed in previous studies (Heapy et al., 2018; Overmayer and Driller, 2018; Oliver and Driller, 2021) and 2) the applied pressure at 135 mmHg is highest between the pre-programmed and commercially available settings in BOOST. In the SQUEE compression, the most distal chamber covering the high ankle to toes inflated for approximately 10 s, after which pressure was held constant to prevent backflow. The same process occurred in the next proximal chamber. This continued until the most proximal chamber (lower thighs) was reached. After inflation was completed in all chambers, they were completely deflated. This entire cycle of compression (approximately 39 s; 1.5 impulses per min) was repeated continuously over the course of a single 30-min treatment session. The SQUEE compression was employed in previous studies (Haun et al., 2017a; 2017b; Oliver and Driller, 2021). In the BOOST compression, all chambers inflated to target pressures for approximately 16 s, and all chambers incompletely deflated for approximately 1 s (approximately 45 mmHg and 8 mmHg at the most distal and proximal chambers, respectively). This was repeated for three times and then all chambers were completely deflated for approximately 9 s. This entire cycle of compression (approximately 60 s; three impulses per min) was repeated continuously over the course of a 30-min treatment session. The frequency at the BOOST mode was the highest among commercially available settings.

The SHAM consisted of wearing the IPC devices and connection to the pneumatic pump but was devoid of actual compression. This trial was used to control any thermogenic effect of the application of IPC devices, as heat loss from the legs is likely affected.

#### 2.5 Measurements

##### 2.5.1 Ankle DF ROM

Ankle DF ROM in the dominant leg was measured using the Weight-Bearing Lunge Test (WBLT) before (Pre), immediately after IPC treatment (Post-0), and 20 min after IPC treatment (Post-20). High inter-rater (r = 0.99) and intra-rater (r = 0.98) reliabilities have been previously reported for the WBLT (Bennell et al., 1998), and ankle DF ROM during the WBL has been a more sensitive measure for identifying those with high-risk movement patterns for injury compared with non-WBL passive measures (Dill et al., 2014). In brief, the participants stood upright against the wall and placed the dominant leg next to a tape measure placed perpendicular to the wall. The participants attached the front of the knee of the dominant leg to the wall while keeping the heel firmly planted on the floor. The involved limb progressed away from the wall while maintaining knee contact without lifting the heel. To ensure that no elevation of the heel occurred, a TheraBand was placed under the heel, and tension was applied by the same experimenter. The TheraBand would snap back if the heel came off the floor, and the distance from the wall to the toe was measured.

##### 2.5.2 Physiological index

The time-averaged mean blood flow velocity (V_mean_) and vessel diameter in the popliteal artery were measured in the dominant leg at Pre, Post-0, and Post-20 using ultrasonography with Doppler and B-mode functions (JS2, Medicare, Japan) with a 4–16 MHz linear transducer and proprietary software (JS2). The V_mean_ was measured with the probe appropriately positioned to maintain an insonation angle of 60° or less. The sample volume was maximized according to the vessel size. Using arterial diameter and V_mean_, blood flow in the popliteal artery as an index of hemodynamics was calculated as follows: blood flow = V_mean_ · π (vessel diameter/2)^2^ · 60.

PPT at the calf in the dominant leg as an index of pain sensitivity was measured using an algometer (MF-129AA, JTECH Medical, United States) at Pre, Post-0, and Post-20. The 1.0-cm^2^ probe of the algometer was placed into the gastrocnemius muscle belly at the position of the proximal 30% of the line connecting the popliteal line and the ankle joint lateral malleolus (Inami et al., 2017). The graded force was applied at a constant rate of 50–60 kPa per second until participants verbally reported the presence of pain.

Gastrocnemius muscle hardness as an index of objective relaxation of muscle was measured using an ultrasonography with a strain elastography function (JS2, Medicare, Japan) at Pre, Post-0, and Post-20. An acoustic coupler (EZU-TECPL1, Hitachi Aloka Medical, Japan, elastic modulus: 22.6 kPa) was used as the reference material and was placed between the transducer and the skin. Ultrasonography gel was applied between the transducer and coupler, and between the coupler and skin. The strain ratio was calculated as: strain ratio = strain ratio in the target muscle/strain ratio in the coupler (Yanaoka et al., 2021). Two 6-s video clips were recorded at each measurement point, and the average value of six randomly chosen images was calculated.

Heart rate variability (HRV) as an index of parasympathetic activation was measured using a wearable heart rate sensor (WHS-1, Union Tool, Japan) for 2 min at Pre, Post-0, and Post-20 in the supine position (Arikawa et al., 2020). It is generally recommended for HRV that analysis be performed on a recording at least 2 min (Smith et al., 2013). During the measurement, the respiratory rate was maintained at 15 breaths/min. For the time-domain analysis, the mean heart rate (HR), SD of normal-to-normal interval (SDNN), and square root of the mean squared differences of successive normal-to-normal intervals (RMSSD) were measured. For the frequency-domain analysis, the high-frequency component (HF, 0.15–0.40 Hz), low-frequency component (LF, 0.04–0.15 Hz), and ratio of LF to HF (LF/HF) were calculated. The natural logarithm (Ln) values were calculated for LF and HF.

##### 2.5.3 Perceptual index

Perceived relaxation was measured using a visual evaluation scale (VAS) before, Post-0, and Post-20 (Wood et al., 2021). The VAS scores ranged from 0 to 10, with 0 representing no relaxation and 10 representing the most relaxed state.

#### 2.6 Statistical analysis

The Shapiro–Wilk test was used to check for normality of distribution. Differences between trial, time, and trial × time for the changes in all variables from Pre (Δ) were analyzed using linear mixed models. This analysis was preferred because it allows for missing data, can accurately model different covariate structures for repeated-measures data, and can model between-subject variability (Vandenbogaerde and Hopkins, 2010). Where significance was found, the values were subsequently analyzed using the Bonferroni multiple comparison test. A pseudo-R^2^ is reported as a measure of global effect size in linear mixed models, and we used the predicted score for each participant in the sample, calculated the correlation between the observed and predicted scores, and squared that correlation (Roldan-Valadez et al., 2018). A pseudo-R^2^ was classified as small (0.10–0.29), moderate (0.30–0.49), and large (≥0.5) (Cohen, 1988). Cohen’s d effect size was also presented where necessary, whereby >2.0 was categorized as a very large effect, 1.2–2.0 as a large effect, 0.6–1.2 as a moderate effect, and 0.2–0.6 as a small effect (Hopkins et al., 2009). Statistical significance was set at *p* < 0.05. Statistical analysis was performed using the SPSS software (version 26.0, SPSS Japan, Japan). All values are shown as the mean ± SD.

## 3 Results

All mean values are presented in Supplementary Table S1. The global effect sizes (pseudo-R^2^ correlation between the observed and predicted scores) were 0.44 for ΔWBLT performance, 0.27 for Δblood flow, 0.32 for ΔV_mean_, 0.06 for Δvessel diameter, 0.18 for ΔPPT, 0.07 for Δmuscle hardness, 0.19 for ΔHR, 0.09 for ΔSDNN, 0.11 for ΔRMSSD, 0.13 for ΔLnHF, 0.14 for ΔLnLF, 0.13 for ΔLnLF/LnHF, and 0.25 for Δperceived relaxation.

### 3.1 Ankle DF ROM

**FIGURE 2:**
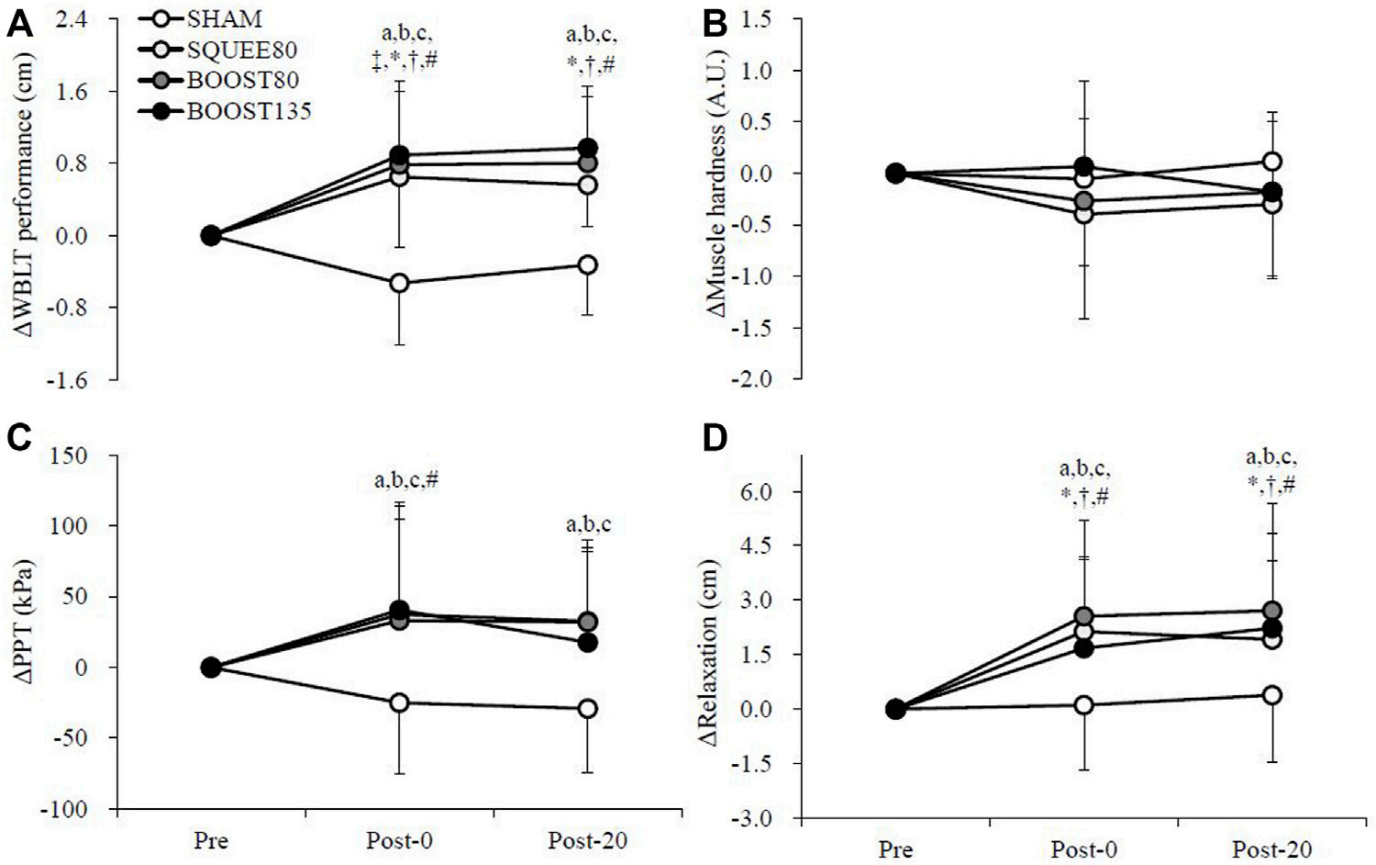
Changes in the Weight-Bearing Lunge Test [WBLT: **(A)**], pressure-to-pain threshold [PPT: **(B)**], muscle hardness **(C)**, and perceived relaxation **(D)** among all trials. Mean ± SD, n = 14. a: SHAM vs. SQUEE80 *p* < 0.05, b: SHAM vs. BOOST80 *p* < 0.05, c: SHAM vs. BOOST135 *p* < 0.05, ‡: vs. Pre in SHAM *p* < 0.05, *: vs. Pre in SQUEE80 *p* < 0.05, †: vs. Pre in BOOST80 *p* < 0.05, # vs. Pre in BOOST135 *p* < 0.05.

Figure 2A shows the changes in WBLT performance. There was a trial × time interaction (F [6,143] = 6.364, *p* = 0.001). ΔWBLT performances in all IPC treatments were significantly higher than SHAM at Post-0 (SQUEE80: *p* < 0.001, d = 1.62, BOOST80: *p* < 0.001, d = 1.75, BOOST135: *p* < 0.001, d = 1.88) and Post-20 (SQUEE80: *p* < 0.001, d = 1.73, BOOST80: *p* < 0.001, d = 1.72, BOOST135: *p* < 0.001, d = 2.05). In all PC treatments, ΔWBLT performances at Post-0 and Post-20 significantly increased compared to those at Pre (vs. Post-0; SQUEE80: *p* = 0.005, d = 1.18; BOOST80: *p* < 0.001, d = 1.36; BOOST135: *p* < 0.001, d = 1.54, vs. Post-20; SQUEE80: *p* = 0.019, d = 1.74; BOOST80: *p* = 0.001, d = 1.54; BOOST135: *p* < 0.001, d = 1.98). In SHAM, ΔWBLT performances at Post-0 significantly decreased compared to that at Pre (*p* = 0.030, d = 1.10).

### 3.2 Popliteal artery blood flow

**FIGURE 3:**
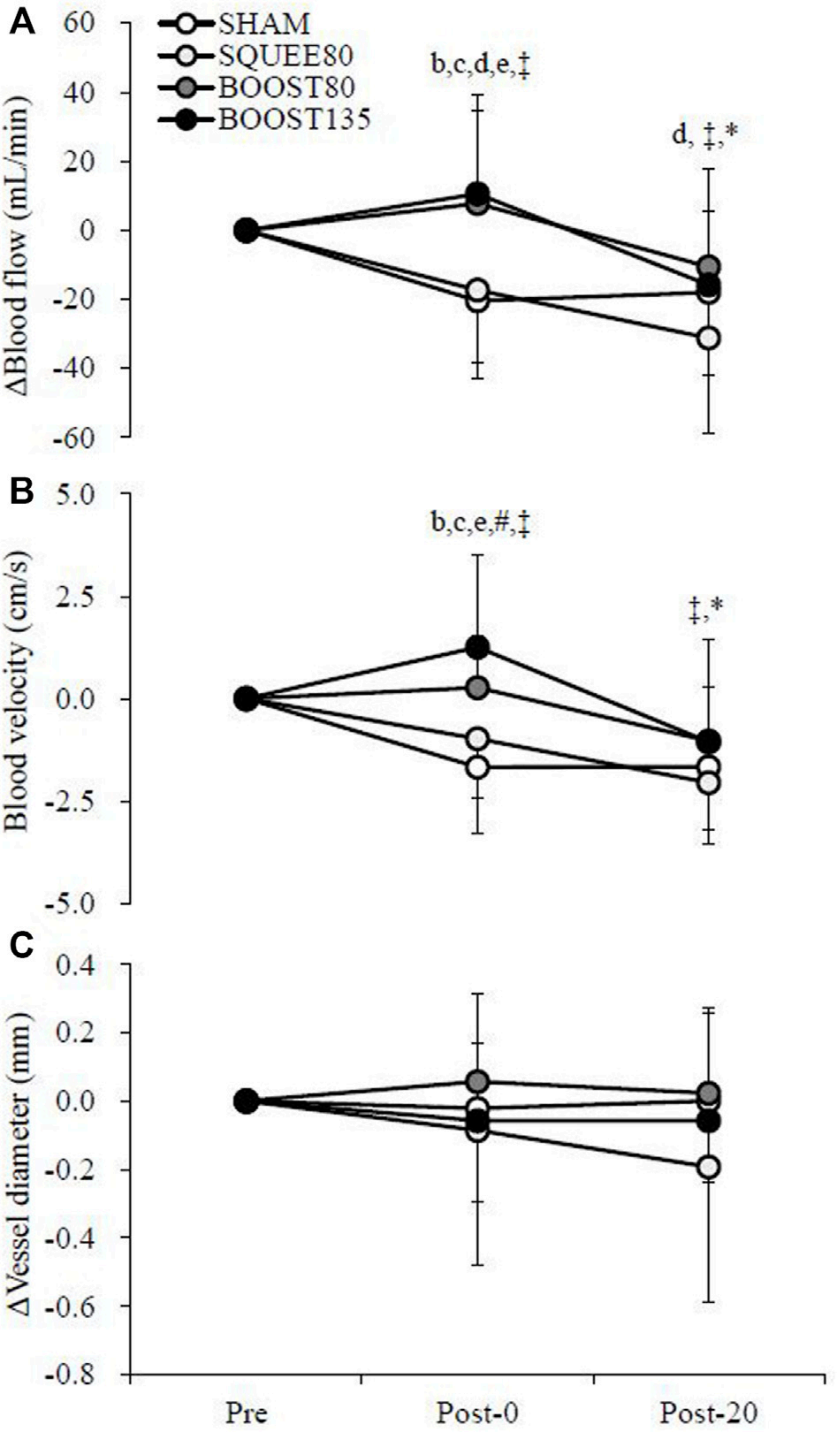
Changes in the blood flow **(A)**, blood velocity **(B)**, and vessel diameter **(C)** of the popliteal artery among all trials. Mean ± SD, n = 14. b: SHAM vs. BOOST80 *p* < 0.05, c: SHAM vs. BOOST135 *p* < 0.05, d: SQUEE80 vs. BOOST80 *p* < 0.05, e: SQUEE80 vs. BOOST135, ‡: vs. Pre in SHAM *p* < 0.05, *: vs. Pre in SQUEE80 *p* < 0.05, # vs. Pre in BOOST135 *p* < 0.05.

Figure 3 shows the changes in the blood flow in the popliteal artery. There were trial × time interactions for Δblood flow (F [6,142] = 3.050, *p* = 0.008) and ΔV_mean_ (F [6,142] = 4.024, *p* = 0.001). ΔBlood flow in BOOST80 and BOOST135 was significantly higher than SHAM and SQUEE80 at Post-0 (vs. SHAM; BOOST80: *p* = 0.001, d = 1.14; BOOST135: *p* < 0.001, d = 1.20, vs. SQUEE80; BOOST80: *p* = 0.005, d = 1.05; BOOST135: *p* = 0.001, d = 1.11). ΔBlood flow in BOOST80 was significantly higher than SQUEE80 at Post-20 (*p* = 0.039, d = 0.91). In SHAM, Δblood flow at Post-0 and Post-20 significantly decreased compared to that at Pre (Post-0: *p* = 0.018, d = 1.29, Post-20: *p* = 0.044, d = 1.07). In SQUEE80, Δblood flow at Post-20 significantly decreased compared to that at Pre (*p* < 0.001, d = 1.60).

ΔV_mean_ in BOOST80 was significantly higher than SHAM at Post-0 (*p* = 0.001, d = 1.34). ΔV_mean_ in BOOST135 was significantly higher than SHAM and SQUEE80 at Post-0 (vs. SHAM; *p* < 0.001, d = 1.50, vs. SQUEE80; *p* < 0.001, d = 1.19). In SHAM, ΔV_mean_ at Post-0 and Post-20 significantly decreased compared to that at Pre (Post-0: *p* = 0.002, d = 1.45, Post-20: *p* = 0.002, d = 1.53). In SQUEE80, ΔV_mean_ at Post-20 significantly decreased compared to that at Pre (*p* < 0.001, d = 1.96). In BOOST135, ΔV_mean_ at Post-0 significantly increased compared to that at Pre (*p* = 0.031, d = 0.80). Owing to missing data, hemodynamics data at Post-20 in BOOST80 were analyzed for 13 participants.

### 3.3 PPT

Figure 2B shows the changes in PPT. There was a trial × time interaction for ΔPPT (F [6,142] = 2.283, *p* = 0.039). ΔPPTs in all IPC treatments were significantly higher than SHAM at Post-0 (SQUEE80: *p* = 0.002, d = 0.94, BOOST80: *p* = 0.004, d = 0.94, BOOST135: *p* = 0.001, d = 1.05) and Post-20 (SQUEE80: *p* = 0.002, d = 1.19, BOOST80: *p* = 0.002, d = 1.24, BOOST135: *p* = 0.032, d = 0.84). In BOOST135, ΔPPT at Post-0 significantly increased compared to that at Pre (*p* = 0.048, d = 0.79). Owing to missing data, PPT data at Post-20 in BOOST135 were analyzed for 13 participants.

### 3.4 Muscle hardness and HRV

Figure 2C shows the changes in gastrocnemius muscle hardness, and Table 1 shows the changes in the HRV data. There were no trial × time interactions for Δmuscle hardness and ΔHRV (*p* > 0.05, Figure 2C and Table 1). Owing to missing data, HRV data were presented for 13 participants.

**TABLE 1:** Mean Δvalues for heart rate variability.

		SHAM			SQUEE80			BOOST80			BOOST135		

ΔHR (bpm)													
	Pre	0.0	±	0.0	0.0	±	0.0	0.0	±	0.0	0.0	±	0.0
	Post-0	-1.5	±	6.2	-1.2	±	4.5	-1.9	±	3.2	-2.5	±	5.0
	Post-20	-1.8	±	4.9	-3.0	±	5.1	-0.7	±	2.7	-3.0	±	3.9
ΔSDNN (ms)													
	Pre	0.0	±	0.0	0.0	±	0.0	0.0	±	0.0	0.0	±	0.0
	Post-0	1.8	±	17.5	4.4	±	21.6	16.1	±	29.4	6.9	±	16.7
	Post-20	19.0	±	17.7	8.9	±	34.6	19.3	±	17.1	5.7	±	30.6
ΔRMSSD (ms)													
	Pre	0.0	±	0.0	0.0	±	0.0	0.0	±	0.0	0.0	±	0.0
	Post-0	-6.1	±	22.1	1.3	±	14.9	8.7	±	22.9	6.7	±	19.7
	Post-20	5.6	±	15.1	7.8	±	22.1	6.2	±	23.7	5.6	±	35.0
ΔLnHF (A.U.)													
	Pre	0.00	±	0.00	0.00	±	0.00	0.00	±	0.00	0.00	±	0.00
	Post-0	-0.54	±	1.20	0.08	±	1.10	0.13	±	0.47	0.12	±	0.90
	Post-20	-0.29	±	0.78	-0.19	±	0.79	0.06	±	0.50	0.07	±	1.08
ΔLnLF (A.U.)													
	Pre	0.00	±	0.00	0.00	±	0.00	0.00	±	0.00	0.00	±	0.00
	Post-0	0.27	±	0.56	0.24	±	0.61	0.19	±	0.68	-0.02	±	0.54
	Post-20	0.45	±	0.71	0.15	±	0.70	0.28	±	0.62	-0.06	±	0.62
ΔLnLF/HF (A.U.)													
	Pre	0.00	±	0.00	0.00	±	0.00	0.00	±	0.00	0.00	±	0.00
	Post-0	0.10	±	0.21	0.03	±	0.06	0.03	±	0.08	0.00	±	0.09
	Post-20	0.08	±	0.12	0.03	±	0.09	0.03	±	0.09	0.00	±	0.12

Mean ± SD, n = 13. HR: heart rate, SDNN: standard deviation of normal-to-normal interval, RMSSD: square root of the mean squared differences of successive normal-to-normal intervals, LnHF: natural logarithm high-frequency component, LnLF: natural logarithm low-frequency component, LF/HF: ratio of LnLF to LnHF.

### 3.5 Perceived relaxation

Figure 2D shows the changes in perceived relaxation. There was a trial × time interaction for Δperceived relaxation (F [6,143] = 2.587, *p* = 0.021). ΔPerceived relaxations in all IPC treatments were significantly higher than SHAM at Post-0 (SQUEE80: *p* = 0.001, d = 1.05, BOOST80: *p* < 0.001, d = 1.08, BOOST135: *p* = 0.022, d = 0.73) and Post-20 (SQUEE80: *p* = 0.028, d = 0.76, BOOST80: *p* < 0.001, d = 0.95, BOOST135: *p* = 0.004, d = 0.81). In all PC treatments, ΔPerceived relaxations at Post-0 and Post-20 significantly increased compared to those at Pre (vs. Post-0; SQUEE80: *p* = 0.001, d = 1.48; BOOST80: *p* < 0.001, d = 1.37; BOOST135: *p* = 0.006, d = 0.96, vs. Post-20; SQUEE80: *p* = 0.005, d = 1.25; BOOST80: *p* < 0.001, d = 1.30; BOOST135: *p* < 0.001, d = 1.20).

## 4 Discussion

This study aimed to investigate the effect of different IPC stimuli on ankle DF ROM. The present findings demonstrate that, in accordance with one of our hypotheses, all IPC treatments significantly increased ankle DF ROM at Post-0 and Post-20, as compared with SHAM. Moreover, the present study showed that all IPC treatments increased PPT at Post-0 and Post-20 compared to SHAM. This suggests that one of the mechanisms of increased ankle DF ROM is an increase in PPT, at least in the present study. These findings support previous findings regarding IPC and flexibility (Sands et al., 2014, 2015; Haun et al., 2017b) and contribute to the growing body of evidence supporting recommendations regarding the use of IPC to increase flexibility. Moreover, to our knowledge, the present study is the first to compare how different IPC stimuli affect ankle DF ROM. No significant differences in WBLT performance at Post-0 and Post-20 were found between all IPC treatments, suggesting that high inflation pressure and frequency may not provide an additional benefit to increase ankle DF ROM, as opposed to our hypotheses.

A decrease in ankle DF ROM has been reported as a predisposing factor for increasing the risk of hamstring strain and ankle injuries (van Dyk et al., 2018), ACL ruptures (Fong et al., 2011), and Achilles and patellar tendinopathies (Backman and Danielson, 2011) in several intermittent team sports with high-intensity stretch-shortening cycles (e.g., soccer and basketball) (Witvrouw et al., 2004). Moreover, ankle DF ROM is critically important in multidirectional running tasks, unilateral dynamic balance, and changes in direction capability (Gonzalo-Skok et al., 2015; López-Valenciano et al., 2019). However, ankle DF ROM throughout the competitive season in professional intermittent team sports is reduced because of the high demand to perform sudden accelerations and decelerations, changes in direction, and jumping and landing tasks (Moreno-Pérez et al., 2020). For instance, Moreno-Pérez et al. reported a significant reduction in WBLT performance in dominant (-9.6%) and non-dominant (-13.8%) ankles from pre-season to post-season (Moreno-Pérez et al., 2020). The present study showed significant increases in WBLT performance in SQUEE80 (+6.1%), BOOST80 (+7.4%), and BOOST135 (+8.2%) at Post-0. Thus, an improvement in ankle DF ROM can be achieved by IPC treatment, which may contribute to avoiding injuries and maintaining exercise performance in sports involving high-intensity stretch-shortening cycles. Although the present study cannot conclude the chronic effects of IPC treatments on exercise performance and risks of injury, it would be interesting to determine whether long-term IPC treatments would have implications for improving exercise performance and injury prevention for intermittent team sports athletes.

Theoretically, the potential mechanisms contributing to increased ROM following massage are a reduction in pain sensitivity, increased hemodynamics, and activation of the parasympathetic nerve (Behm and Wilke, 2019). In the present study, the hemodynamic and parasympathetic aspects were assessed using blood flow, HRV, and muscle hardness. The present study showed 1) no improvement in blood flow in SQUEE80 and 2) no affecting HRV and muscle hardness by employing IPC treatments. Thus, at least in the present study, these may not be the main mechanisms contributing to increased ROM following IPC treatment. However, the present study demonstrated that all IPC treatments increased PPT as compared with SHAM. The reduction in pain sensitivity observed in the present study may be related to the gate control theory of pain (Moayedi and Davis, 2013) and diffuse noxious inhibitory control (Chaillet et al., 2014). Massage stimulates a variety of mechanoreceptors and nociceptors, which can alter the transmission of ascending nociceptive information *via* small-diameter Aδ fibers and give rise to a descending inhibitory effect that facilitates pain inhibition (Moayedi and Davis, 2013). Massage also triggers an endorphinergic system, which reduces pain everywhere except in the stimulated area (Chaillet et al., 2014). Reduction in pain sensitivity and resultant increase in flexibility following IPC treatment are consistent with previous findings reported by other investigators (Sands et al., 2014, 2015; Haun et al., 2017b).

In addition, the present study observed no difference between the IPC treatments in the magnitude of increases in PPT and flexibility. Massage does not need to be unduly painful to decrease pain sensitivity. A previous study suggested that pain inhibition occurs after light rolling massage (Aboodarda et al., 2015). Yanaoka et al. also found that the intensity of rolling massage did not affect the magnitude of the increase in ROM (Yanaoka et al., 2021). Mechanoreceptors and nociceptors are present in both muscle and skin; thus, even light stimulation can decrease pain sensitivity (Behm and Wilke, 2019). Thus, in the present study, no differences in pain sensitivity between IPC treatments may contribute to a similar increase in flexibility following all IPC treatments.

IPC treatments have been widely employed in clinical settings to increase lower-limb artery hemodynamics, resulting in the prevention of deep venous thrombosis and reduction of both lymphedema and venous ulcer healing times (Kumar and Walker, 2002). In addition, an increase in blood flow following IPC potentiates greater oxygenation (Helmi et al., 2014) and nutrient delivery (e.g., blood glucose and plasma insulin) (Keck et al., 2015) and metabolite clearance (e.g., blood lactate) (Martin et al., 2015b) after exercise, which is intriguing in the context of athletes’ recovery. Although popliteal artery blood flow during IPC treatments could not be measured because of the encapsulating nature of the leg sleeves, the present study revealed that popliteal artery blood flow at Post-0 in SHAM and SQUEE80 was decreased by 16.2% and 12.4%, respectively, from Pre; whereas this decrease was significantly prevented by BOOST80 (+5.8%) and BOOST135 (+7.8%). Decrements in arterial blood flow at Post-0 in SHAM and SQUEE80 are consistent with previous findings suggesting lower resting limb blood flow after a 1-h IPC treatment (squeeze method, ∼70 mmHg) and sham (wearing IPC devices without compression) (Martin et al., 2015a). In addition, the present study is the first to observe significant prevention of decreased arterial blood flow at Post-0 in BOOST80 and BOOST135. Previous studies reported that sequential compressions may have a greater and more sustained increase in lower-limb hemodynamics than uniform compressions [see the previous review of these, (Helmi et al., 2014)]; thus, the present result is inconsistent with previous findings. This may be due to the differences in compression frequencies between SQUEE (1.5 impulses per min) and BOOST (three impulses per min). One of the mechanisms of improved arterial blood flow following IPC treatment is an increase in arteriovenous pressure gradient secondary to venous emptying (Delis et al., 2000, 2001). Delis et al. investigated the optimum IPC stimulus for venous emptying and suggested that a frequency of three impulses per min generated significantly higher venous emptying than a frequency of two impulses per min at applied pressures of 60–140 mmHg when IPC was applied at both the foot and calf (Delis et al., 2000). A frequency of three impulses per min can provide refilling that starts at the end of compression because the venous refill time is approximately 20 s (Delis et al., 2000). This suggests that the frequency of 1.5 impulses per min used in the present study may be insufficient to maintain empty veins. Thus, compression frequency may contribute to a significant prevention of decreased arterial blood flow at Post-0 in BOOST80 and BOOST135.

A previous study reported that IPC treatment suppresses parasympathetic activation, as evidenced by decreased HF (Valenzuela et al., 2018). Increased parasympathetic activation can contribute to decreased pain sensitivity, increased muscle relaxation, and increased hemodynamics (Weerapong et al., 2005); thus, the context of recovery for athletes is critical. The present study demonstrated that IPC treatment had no influence on HRV, which is inconsistent with a previous study (Valenzuela et al., 2018). This may be due to the difference in compression sites between the present and previous studies (Valenzuela et al., 2018). The previous study employed abdominal compression (Valenzuela et al., 2018). Bladder distension has been clearly demonstrated in human sympathetic activation (Fagius and Karhuvaara, 1989). Indeed, abdominal compression by pneumatic anti-shock garments activated the sympathetic nervous system as compared to limb compression (Garvin et al., 2014).

Despite the insights provided by this study, there are some limitations that need to be considered. First, the present study cannot conclude the recovery effects of IPC treatment on ankle DF ROM. IPC treatments are likely employed after exercise to accelerate recovery (Overmayer and Driller, 2018). Further research is needed to investigate how IPC treatment affects ankle DF ROM recovery. Second, popliteal artery blood flow was not measured during IPC treatment. It is reasonable to assume that the popliteal artery blood flow increases during IPC treatment (Delis et al., 2000, 2001). It would be interesting to determine whether the different IPC stimuli used in the present study would have similar effects on increases in popliteal artery blood flow. Third, the interpretation of results in ankle DF ROM must be acknowledged. Although IPC treatments increase flexibility, this is likely to promote better performances and decreases the risk of injuries in only sports with a high intensity of stretch-shortening cycles (Witvrouw et al., 2004). In sports containing low-intensity or limited stretch-shortening cycles (e.g., jogging, cycling, and swimming), increased flexibility may not be advantageous (Witvrouw et al., 2004). Finally, we could not compare between SQUEE at 135 mmHg and the other IPC treatments since SQUEE at 135 mmHg did not pre-program commercially.

In conclusion, this study demonstrated that IPC treatment, irrespective of applied pressure and mode of compression, increased ankle DF ROM due to an increase in PPT. Additional benefits of high pressure and frequency were not observed for the increase in ankle DF ROM. In addition, IPC treatments in BOOST increased popliteal artery blood flow immediately after IPC compared to SHAM and SQUEE.

### 4.1 Practical applications

This study revealed that IPC treatments, irrespective of applied pressure and mode of compression, increased ankle DF ROM; however, blood flow at Post-0 was higher after high-frequency IPC treatments than that with low-frequency treatments. These suggested that IPC treatments with high frequency may promote recovery for athletes. In addition, IPC can be mixed with other recovery modalities, such as hydration and nutritional strategies. When athletes must play in several twilight matches per week (e.g., FIFA World Cup in football, Olympic games in basketball), maximizing recovery within a short amount of time becomes critical. The accumulation and combination of several recovery techniques would support maximal recovery; thus, IPC treatments, which can be easily performed with other recovery modalities, would be recommended for athletes.

## Data Availability

The raw data supporting the conclusions of this article will be made available by the authors, without undue reservation.
